# Supplementary material for: Evaluation of Long-Term Performance of Six PM2.5 Sensor Types
Source: Sensors (Basel). 2025 Feb 19;25(4):1265. doi: 10.3390/s25041265 (PMC11861664; doi:10.3390/s25041265)
Supplement: Supplementary file 1 [file sensors-25-01265-s001.zip › sensors-3412783-supplementary.pdf]

1 Supplement

1.0 Additional Sensor details

Table S 1. LTPP Sensor List with additional details. Many of the temperature and RH measurements may be internal measurements instead of ambient measurements. Some of the sampling intervals could be modified by the user or by the manufacturer, but these were the intervals used during this project.

Device Name	Device Code	Measured Pollutants	Sampling Interval	Firmware version
AQY	AQY	$PM_{2.5}$, NO_2, O_3, T, RH	1 min	V1.14.2
Clarity Node/ Node-S	CNO	$PM_{2.5}$, NO_2*, T, RH	~5 min *(Node)*<br>~15 min *(Node-S, NC only)*	Received by EPA May 2019†
Maxima	MAX	PM_1, $PM_{2.5}$, PM_{10}, T, RH, P	30 sec	v5.0
PurpleAir	PAR	PM_1, $PM_{2.5}$, PM_{10}	2 min	v4.02
RAMP	RAM	$PM_{2.5}$, CO, NO, NO_2, SO_2, O_3	15 sec	181023_AQ_v9.18
ARISense	ARS	PM_1, $PM_{2.5}$, PM_{10}, CO, CO_2, NO, NO_2, O_3, T, RH, P, WS, WD	2 min	Received by EPA April 2019†

* Clarity discontinued reporting NO_2 concentration in the data output for the LTPP units in late August 2019.

1.0.1 AQY

The Aeroqual AQY is a multi-pollutant sensor pod measuring T, RH, dew point, NO_2, O_3, and $PM_{2.5}$. The AQY uses the Aeroqual gas sensitive semiconductor sensor to both measure O_3 and to correct for the O_3 interference on the Aeroqual electrochemical NO_2 sensor. PM is measured using a NOVA SDS011 sensor and T, RH, and dew point are measured using a digital sensor module. The AQY can transmit data over Wi-Fi, or via cellular signal using a pre-installed modem and subscriber identification module (SIM) card. For this study, SIM cards were obtained from Aeroqual and installed in all sensors except those deployed in NC. The devices operating in NC at the Air Innovation Research Site (AIRS) were connected to Wi-Fi via a mobile hotspot installed at the site. The 1-minute raw data were acquired weekly using the Aeroqual Cloud user interface (UI) (cloud.aeroqual.com). The AQY possesses an internal data storage USB flash drive as a data backup, however the software on the drive is proprietary to Aeroqual and unreadable by outside systems. We did not use Aeroqual's calibration feature where users can input a slope and offset into the online dashboard after conducting an initial colocation.

Table S 2. Aeroqual deployment locations and time periods by serial IDs. Sensors were first colocated in NC (deploy=Pre) before being sent for ~1-year colocation (deploy=deploy) and then a final colocation in NC (deploy=Post).

Location	serial_id	deploy	start	end
NC	BA-317A	deploy	8/1/2019 12:00	1/1/2021 4:00
NC	BA-317A	Post	6/2/2021 5:00	7/6/2021 5:00
NC	BA-317A	Pre	5/10/2019 1:00	6/8/2019 14:00
WI	BA-318A	deploy	7/24/2019 6:00	12/22/2020 1:00
NC	BA-318A	Post	6/2/2021 5:00	7/6/2021 5:00
NC	BA-318A	Pre	5/14/2019 20:00	6/8/2019 14:00
AZ	BA-319A	deploy	8/15/2019 7:00	12/9/2020 17:00
NC	BA-319A	Post	6/2/2021 5:00	7/6/2021 5:00
NC	BA-319A	Pre	5/10/2019 1:00	5/24/2019 20:00
NC	BB-522	deploy	8/1/2019 12:00	1/1/2021 4:00
NC	BB-522	Pre	6/21/2019 1:00	6/26/2019 13:00
NC	BB-523	deploy	8/1/2019 12:00	9/21/2020 19:00
NC	BB-523	Pre	6/21/2019 1:00	6/26/2019 13:00
CO	BB-524	deploy	7/31/2019 18:00	1/1/2021 6:00
NC	BB-524	Post	6/2/2021 5:00	6/3/2021 22:00
NC	BB-524	Pre	6/21/2019 1:00	6/26/2019 13:00
DE	BB-525	deploy	7/26/2019 18:00	1/1/2021 3:00
NC	BB-525	Post	6/2/2021 9:00	7/1/2021 19:00
NC	BB-525	Pre	6/21/2019 1:00	6/26/2019 13:00
GA	BB-526	deploy	8/1/2019 17:00	1/1/2021 4:00
NC	BB-526	Post	6/2/2021 5:00	7/6/2021 5:00
NC	BB-526	Pre	6/21/2019 1:00	6/26/2019 13:00
OK	BB-527	deploy	8/1/2019 17:00	1/1/2021 5:00
NC	BB-527	Post	6/2/2021 5:00	7/6/2021 5:00
NC	BB-527	Pre	6/21/2019 1:00	6/26/2019 13:00

1.0.2 ARISense

The ARISense (ARS) is an ambient environmental monitoring unit containing an ParticlePlus Optical Particle Counter (OPC) for detection of PM and a multi-pollutant measurement system. The OPC can measure PM in a diameter range between 0.4 and 17 microns with multiple size bins. The sensor estimates PM_1, $PM_{2.5}$ and PM_{10} mass concentrations (µg/m^3). The multi-pollutant measurement system includes electrolytic sensors that measure gas phase pollutants: NO, NO_2, CO, and total oxidants (O_3 + NO_2), as well as a Non-Dispersive Infrared (NDIR) sensor to measure CO_2. The ARS also comes with optional peripheral sensors to measure the following environmental and meteorological parameters: RH, T, solar intensity, pressure (P), ambient noise, windspeed (WS) and wind direction (WD). The ARS uploads data to an external cloud server at 2-minute averages. The ARS weekly data collections were performed over Wi-Fi using the ARS QuantAQ cloud server (https://www.quat-aq.com). A final dataset with a correction applied was provided by the manufacturer.

Table S 3. ARISense deployment locations and time periods by serial IDs. Sensors were first colocated in NC (deploy=Pre) before being sent for ~1 year of colocation (deploy=deploy) and then a final colocation in NC (deploy=Post).

Location	serial_id	deploy	start	end
NC	SN000-039	deploy	11/23/2019 4:00	12/31/2019 23:00
OK	SN000-039	deploy	2/28/2020 23:00	6/3/2020 9:00
NC	SN000-039	Pre	3/27/2019 21:00	4/9/2019 18:00
NC	SN000-041	deploy	12/3/2019 21:00	12/31/2019 23:00
NC	SN000-041	Pre	3/27/2019 18:00	6/26/2019 4:00
NC	SN000-042	deploy	12/2/2019 22:00	10/28/2020 23:00
NC	SN000-042	Post	3/28/2021 1:00	4/5/2021 23:00
NC	SN000-042	Pre	3/27/2019 21:00	6/24/2019 23:00
NC	SN000-043	deploy	12/30/2019 20:00	1/3/2021 2:00
NC	SN000-043	Pre	3/27/2019 21:00	6/26/2019 4:00
NC	SN000-044	deploy	12/5/2019 22:00	12/31/2019 23:00
GA	SN000-044	deploy	2/8/2020 13:00	4/30/2020 12:00
NC	SN000-044	Pre	3/27/2019 20:00	6/26/2019 4:00
NC	SN000-047	deploy	12/2/2019 22:00	2/21/2021 15:00
NC	SN000-047	Post	2/21/2021 16:00	4/5/2021 23:00
NC	SN000-047	Pre	3/27/2019 21:00	6/8/2019 14:00
NC	SN000-048	deploy	12/3/2019 21:00	2/20/2021 7:00
NC	SN000-048	Post	3/29/2021 17:00	3/31/2021 19:00
NC	SN000-048	Pre	3/27/2019 20:00	6/26/2019 3:00
NC	SN000-050	deploy	12/7/2019 18:00	12/31/2019 23:00
AZ	SN000-050	deploy	2/7/2020 1:00	12/9/2020 13:00
NC	SN000-050	Pre	3/27/2019 21:00	6/8/2019 14:00
NC	SN000-051	deploy	11/23/2019 4:00	12/31/2019 23:00
CO	SN000-051	deploy	1/1/2021 1:00	2/19/2021 16:00
NC	SN000-051	Pre	3/27/2019 21:00	6/26/2019 3:00
NC	SN000-054	deploy	12/23/2019 16:00	12/29/2020 12:00
NC	SN000-054	Pre	3/27/2019 21:00	6/26/2019 3:00
NC	SN000-056	deploy	12/2/2019 22:00	2/21/2021 15:00
NC	SN000-056	Post	2/21/2021 16:00	4/5/2021 23:00
NC	SN000-056	Pre	3/27/2019 21:00	6/26/2019 3:00
NC	SN000-065	deploy	12/2/2019 22:00	12/27/2020 11:00
NC	SN000-065	Pre	3/27/2019 20:00	6/26/2019 4:00
NC	SN000-070	deploy	11/23/2019 4:00	12/31/2019 23:00
WI	SN000-070	deploy	2/7/2020 1:00	1/28/2021 16:00
NC	SN000-070	Pre	3/27/2019 21:00	6/26/2019 4:00
NC	SN000-071	deploy	12/2/2019 22:00	2/21/2021 15:00
NC	SN000-071	Post	2/21/2021 16:00	4/5/2021 23:00
NC	SN000-071	Pre	3/27/2019 21:00	6/26/2019 4:00
NC	SN000-073	deploy	12/12/2019 14:00	12/29/2020 12:00
NC	SN000-073	Pre	3/27/2019 20:00	6/26/2019 4:00
NC	SN000-074	deploy	11/23/2019 4:00	12/31/2019 23:00
DE	SN000-074	deploy	2/5/2020 18:00	2/21/2021 15:00
NC	SN000-074	Pre	3/27/2019 21:00	6/26/2019 4:00

1.0.3 Clarity Node

The Clarity Node (CNO) reports concentrations of PM from the Plantower PMS6003 in three size ranges: PM_1, $PM_{2.5}$, and PM_{10} (µg/m^3), NO_2 concentration (electrochemical sensor) as well as T and RH. A proprietary algorithm is used to produce an Air Quality Index (AQI) rating based upon the U.S. EPA AQI calculation. The CNO uses a small fan to pull nearby pollutants past the array of sensors. The operable temperature range is between -10 and 55 ◦C with a RH range of 10-90%. The CNO transmits data via cellular SIM card and records data at five or fifteen-minute intervals (according to device manual), which is dependent on the device power option (electric vs. solar). After initial evaluation, it was determined that the sampling interval is dynamic and can increase or decrease depending on user settings, battery charge, and cellular signal strength. On average the Clarity Node sensors ran at 2- to 3-minute intervals with no discernable patterns, while the AIRS devices (Clarity Node-S) ran at 17-minute intervals. Only the devices deployed at the local AIRS field site were solar powered (Node-S) and thus had a larger sampling interval. Weekly data collections were performed over Wi-Fi using the Clarity Smart City UI (https://smartcity.clarity.io/). However, the final dataset was provided by the manufacturer with the new 2021 wildfire correction applied since not all required variables were available in the downloads retrieved during the project.

Table S 4. Clarity deployment locations and time periods by serial IDs. Sensors were first colocated in NC (deploy=Pre) before being sent for ~1 year of colocation (deploy=deploy) and then a final colocation in NC (deploy=Post).

Location	serial_id	deploy	start	end
NC	A1N629Q4	deploy	8/1/2019 12:00	2/21/2021 15:00
NC	A1N629Q4	Post	2/21/2021 16:00	7/6/2021 5:00
NC	A1N629Q4	Pre	6/21/2019 1:00	6/26/2019 13:00
OK	A2GM57YJ	deploy	8/1/2019 16:00	1/1/2021 5:00
NC	A2GM57YJ	Post	6/2/2021 1:00	6/23/2021 10:00
NC	A2GM57YJ	Pre	6/21/2019 1:00	6/26/2019 13:00
NC	A80S13VJ	deploy	7/25/2019 17:00	2/21/2021 15:00
NC	A80S13VJ	Post	2/21/2021 16:00	7/6/2021 5:00
NC	A80S13VJ	Pre	6/21/2019 1:00	6/26/2019 13:00
CO	AC0D8N34	deploy	7/31/2019 18:00	10/2/2020 18:00
NC	AC0D8N34	Pre	6/21/2019 1:00	6/26/2019 13:00
GA	AQ8YWKQ3	deploy	8/1/2019 17:00	1/5/2021 14:00
NC	AQ8YWKQ3	Post	6/2/2021 1:00	7/6/2021 5:00
NC	AQ8YWKQ3	Pre	6/21/2019 1:00	6/26/2019 13:00
DE	AV0B3XWV	deploy	7/26/2019 18:00	10/1/2020 13:00
NC	AV0B3XWV	Post	6/2/2021 1:00	6/24/2021 10:00
NC	AV0B3XWV	Pre	6/21/2019 1:00	6/26/2019 13:00
AZ	AVKVJKTB	deploy	7/26/2019 22:00	10/2/2020 17:00
NC	AVKVJKTB	Post	6/2/2021 1:00	7/6/2021 5:00
NC	AVKVJKTB	Pre	6/21/2019 1:00	6/26/2019 13:00
NC	AWKHL3GR	deploy	8/1/2019 12:00	2/21/2021 15:00
NC	AWKHL3GR	Post	2/21/2021 16:00	7/6/2021 5:00
NC	AWKHL3GR	Pre	6/21/2019 1:00	6/26/2019 13:00
WI	AYSNHJJ8	deploy	7/22/2019 6:00	10/2/2020 18:00
NC	AYSNHJJ8	Post	6/2/2021 1:00	6/16/2021 9:00
NC	AYSNHJJ8	Pre	6/21/2019 1:00	6/26/2019 13:00

Equation S 1. The 2021 Wildfire correction for Clarity data.

CNO_wf =

pm1ConcMass.raw * -0.500717803737932 +

pm2_5ConcMass.raw * -0.265672980527763 +

pm10ConcMass.raw * 0.306926940158569 +

pm1ConcNum.raw * -0.321049535207158 +

pm2_5ConcNum.raw * -2.98685668409478 +

pm10ConcNum.raw * 3.82509799771366 +

relHumid.raw * -0.0510318270483026 +

4.28415022383616

1.0.4 Maxima

The Applied Particle Technologies (APT) Maxima (MAX) is an ambient environmental monitoring device which uses the Plantower PMS A003 PM sensor. The PM sensor reportedly measures particle size distribution ranging from 0.3 µm to >10 µm, with a concentration range between 0 and 1000 µg/m^3. In addition to PM, the MAX also measures T, RH, and P. The operable temperature range is reportedly between 0 and 65 ◦C, with a RH range of 0-100% and a pressure of 300-1100 hPa. The MAX records data at 30-second intervals and stores the data on an internal microSD card and transmits data to the APT cloud server3 via Wi-Fi connection. The user interface (UI) for the MAX (https://appliedparticle.io/) sensor pods was still being developed at the start of this project; thus, weekly raw data files were obtained from the internal MicroSD card. Each field site operator was provided two labeled MicroSD cards for the MAX that they used to swap out each week (i.e. SD swap method). Data from the collected card was then read and processed.

Table S 5 MAX deployment locations and time periods by serial IDs. Sensors were first colocated in NC (deploy=Pre) before being sent for ~1 year of colocation (deploy=deploy) and then a final colocation in NC (deploy=Post).

Location	serial_id	deploy	start	end
NC	MX_B_002	deploy	8/1/2019 12:00	9/15/2019 1:00
WI	MX_B_002	deploy	8/16/2019 13:00	10/2/2020 16:00
NC	MX_B_002	Post	6/2/2021 17:00	7/2/2021 3:00
AZ	MX_B_003	deploy	7/26/2019 22:00	10/3/2020 5:00
NC	MX_B_003	Post	6/2/2021 17:00	7/5/2021 12:00
NC	MX_B_003	Pre	3/15/2019 14:00	6/8/2019 14:00
WI	MX_B_004	deploy	7/17/2019 12:00	8/16/2019 11:00
NC	MX_B_004	deploy	9/4/2019 11:00	2/21/2021 4:00
NC	MX_B_004	Post	6/2/2021 17:00	7/3/2021 22:00
NC	MX_B_004	Pre	3/15/2019 14:00	6/8/2019 14:00
NC	MX_B_009	deploy	8/1/2019 12:00	11/1/2019 13:00
CO	MX_B_009	deploy	8/21/2019 15:00	10/2/2020 20:00
NC	MX_B_009	Post	6/2/2021 17:00	7/4/2021 9:00
NC	MX_B_009	Pre	5/13/2019 21:00	5/30/2019 10:00
CO	MX_B_010	deploy	7/31/2019 18:00	9/4/2019 13:00
NC	MX_B_010	deploy	10/17/2019 14:00	1/30/2021 4:00
NC	MX_B_010	Post	6/11/2021 20:00	7/6/2021 5:00
NC	MX_B_010	Pre	3/15/2019 14:00	6/8/2019 14:00
NC	MX_B_011	deploy	2/12/2020 19:00	2/20/2020 14:00
DE	MX_B_011	deploy	7/26/2019 18:00	10/1/2020 12:00
NC	MX_B_011	Post	6/2/2021 17:00	7/6/2021 5:00
NC	MX_B_011	Pre	3/15/2019 14:00	6/8/2019 14:00
GA	MX_B_012	deploy	8/1/2019 18:00	1/5/2021 14:00
NC	MX_B_012	Post	6/23/2021 15:00	7/4/2021 23:00
NC	MX_B_012	Pre	3/15/2019 14:00	6/8/2019 14:00
NC	MX_B_013	deploy	8/1/2019 12:00	2/10/2021 13:00
NC	MX_B_013	Post	6/2/2021 17:00	7/3/2021 6:00
NC	MX_B_013	Pre	5/13/2019 21:00	6/8/2019 14:00
OK	MX_B_014	deploy	8/1/2019 16:00	12/22/2020 14:00
NC	MX_B_014	Post	6/9/2021 21:00	7/3/2021 7:00
NC	MX_B_014	Pre	3/15/2019 14:00	6/8/2019 14:00

1.0.5 PurpleAir

The PurpleAir PA-II-SD (PAR) uses two PMS5003 laser particle counters to report concentrations of PM1, $PM_{2.5}$, and PM_{10} (µg/m^3), particle counts in several size bins (<0.3µm, <0.5µm, <1µm, <2.5µm, <5µm, and <10µm), as well as T, RH, and P. The PAR records data at a 2-minute sampling interval. Data are transmitted to the PAR Cloud via Wi-Fi and stored on an internal MicroSD card. The data from the PurpleAir ThingSpeak API was used for the analysis in this paper.

Table S 6. PurpleAir deployment locations and time periods by serial IDs. Sensors were first colocated in NC (deploy=Pre) before being sent for ~1 year of colocation (deploy=deploy) and then a final colocation in NC (deploy=Post).

Location	serial_id	deploy	start	end
AZ	de89	deploy	8/1/2019 1:00	10/2/2020 16:00
NC	de89	Pre	10/29/2018 5:00	11/1/2018 4:00
NC	de90	deploy	7/23/2019 17:00	12/31/2020 2:00
NC	de90	Post	6/2/2021 3:00	7/5/2021 8:00
NC	de90	Pre	10/29/2018 5:00	11/1/2018 4:00
CO	de92	deploy	8/1/2019 1:00	10/2/2020 17:00
NC	de92	Post	6/2/2021 1:00	7/6/2021 5:00
NC	de92	Pre	10/29/2018 5:00	11/1/2018 4:00
WI	e9a8	deploy	7/22/2019 20:00	10/2/2020 16:00
NC	e9a8	Post	6/2/2021 1:00	7/6/2021 5:00
NC	e9a8	Pre	10/29/2018 5:00	10/29/2018 15:00
DE	e9ae	deploy	7/29/2019 1:00	10/1/2020 11:00
NC	e9ae	Post	6/2/2021 1:00	7/6/2021 5:00
NC	e9ae	Pre	10/29/2018 5:00	11/1/2018 4:00
NC	e9ba	deploy	7/23/2019 17:00	12/30/2020 20:00
NC	e9ba	Post	6/2/2021 15:00	7/2/2021 20:00
NC	e9ba	Pre	10/29/2018 5:00	10/29/2018 15:00
GA	ec2	deploy	8/2/2019 1:00	1/3/2021 22:00
NC	ec2	Post	6/2/2021 1:00	7/6/2021 5:00
NC	ec2	Pre	10/29/2018 5:00	11/1/2018 4:00
OK	ec6d	deploy	8/2/2019 11:00	12/22/2020 14:00
NC	ec6d	Post	6/2/2021 2:00	7/5/2021 11:00
NC	ec6d	Pre	10/29/2018 5:00	11/1/2018 4:00
NC	ec8	deploy	7/23/2019 17:00	9/2/2020 14:00
NC	ec8	Post	6/2/2021 1:00	7/6/2021 5:00
NC	ec8	Pre	10/29/2018 5:00	11/1/2018 4:00

1.0.6 RAMP

The SenSit RAMP (RAM) is a weatherproof multi-pollutant modular sensor pod that uses a COZIR AH-2000 sensor to measure CO_2, T and RH, the Alphasense Type B4 sensors to measure NO_2, NO, CO, and O_3, and the Plantower PMS5003 sensor to measure PM_1, $PM_{2.5}$, and PM_{10} in ambient air. It can interface with an anemometer as well; however, one was not used for this experiment. RAMP data are stored as daily text files on an internal MicroSD card. This sensor could not be configured to report data at the top of every minute instead reporting approximately every 60 seconds after the unit was powered up. Since our original intention was to, at some point, compare 1-min averaged measurements the sensor was configured to provide data at 15-second intervals so that the data could be averaged to a 1-minute average with less averaging uncertainty. The weekly raw data files were obtained via the SD card swap method.

Table S 7. RAMP deployment locations and time periods by serial IDs. Sensors were first colocated in NC (deploy=Pre) before being sent for roughly a 1 year of colocation (deploy=deploy) and then a final colocation in NC (deploy=Post).

Location	serial_id	deploy	start	end
WI	S1015	deploy	7/22/2019 6:00	10/2/2020 16:00
NC	S1015	Post	6/2/2021 5:00	7/6/2021 5:00
NC	S1015	Pre	4/11/2019 13:00	6/8/2019 14:00
CO	S1016	deploy	8/1/2019 7:00	11/13/2019 14:00
NC	S1016	deploy	2/27/2020 17:00	2/20/2021 23:00
NC	S1016	Post	2/21/2021 18:00	2/24/2021 3:00
NC	S1016	Pre	2/14/2019 16:00	4/9/2019 23:00
DE	S1017	deploy	7/29/2019 5:00	3/12/2020 4:00
NC	S1017	Pre	2/14/2019 16:00	4/9/2019 23:00
DE	S1018	deploy	3/12/2020 5:00	10/1/2020 12:00
NC	S1018	deploy	10/17/2019 13:00	2/27/2020 15:00
NC	S1018	Post	6/2/2021 5:00	7/6/2021 5:00
NC	S1018	Pre	2/14/2019 16:00	4/9/2019 23:00
NC	S1019	deploy	8/1/2019 12:00	8/8/2019 10:00
GA	S1019	deploy	8/15/2019 16:00	12/30/2020 4:00
NC	S1019	Post	6/2/2021 5:00	7/6/2021 5:00
NC	S1019	Pre	2/23/2019 5:00	2/25/2019 8:00
OK	S1020	deploy	8/14/2019 18:00	12/22/2020 14:00
NC	S1020	Post	6/2/2021 5:00	7/6/2021 5:00
NC	S1020	Pre	2/26/2019 19:00	3/1/2019 13:00
NC	S181	deploy	8/1/2019 12:00	1/1/2021 4:00
NC	S181	Post	6/2/2021 5:00	7/6/2021 5:00
NC	S181	Pre	2/14/2019 16:00	4/9/2019 23:00
NC	S182	deploy	8/1/2019 12:00	11/14/2019 12:00
CO	S182	deploy	11/22/2019 7:00	6/10/2020 13:00
NC	S182	Post	6/2/2021 5:00	7/6/2021 5:00
NC	S182	Pre	4/11/2019 13:00	6/8/2019 14:00
AZ	S183	deploy	8/12/2019 8:00	10/2/2020 17:00
NC	S183	Post	6/2/2021 5:00	7/6/2021 5:00
NC	S183	Pre	4/11/2019 13:00	6/7/2019 8:00

1.1 Additional Details on Selected Sites

Sites were selected to encompass a variety of environmental conditions and pollutant mixtures as experienced across the U.S. As much sensor pod evaluation work is being done in California, they were excluded from consideration to focus on areas where less is known about sensor pod performance. Selected sites needed to meet criteria including local agency support and interest in the project, the availability of a T640 $PM_{2.5}$ FEM, for comparison, site access for field technicians or willingness to support the instruments themselves, and power availability with a preference for sites that could offer wireless connectivity. We ended up using a TEOM at the AZ site due to the high concentrations experienced and the known bias of the T640 [15,73,74].

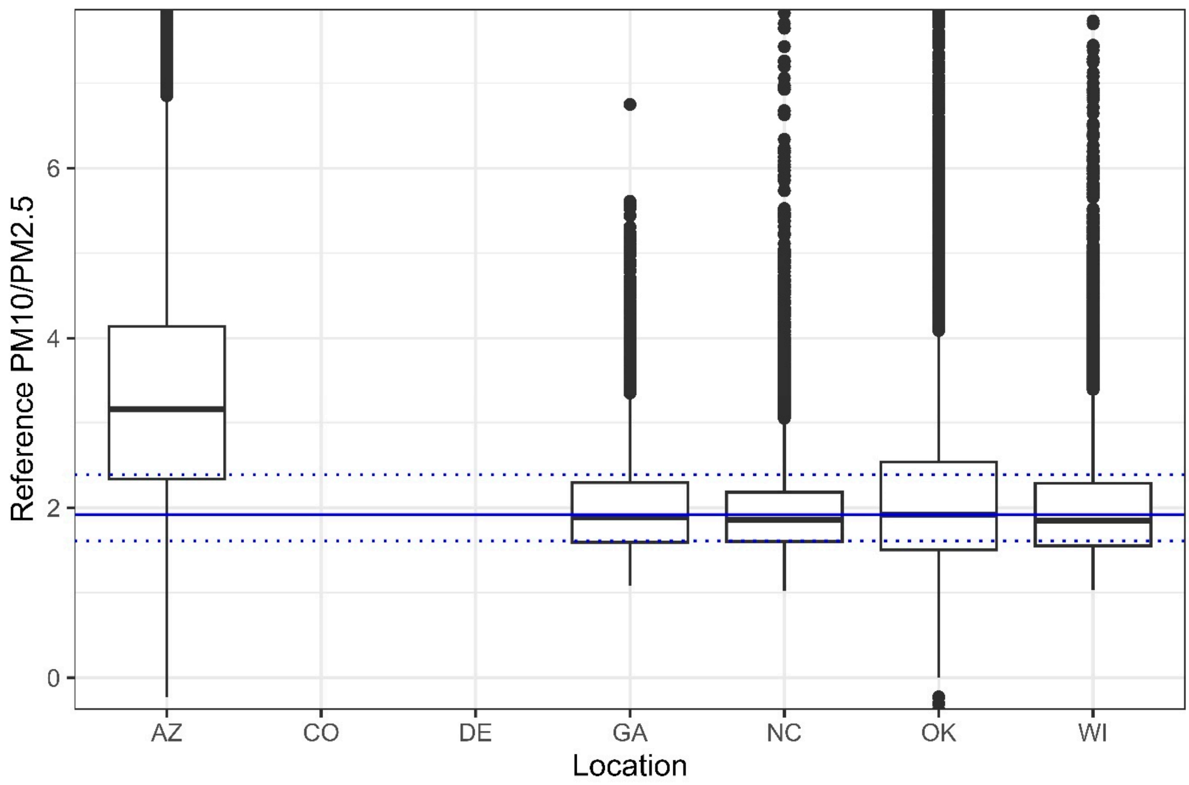

Figure S 1. Reference monitor $PM_{10}/PM_{2.5}$ ratio by location where available. The blue solid line is the median for all sites (1.92) dashed lines are the overall 1st and 3rd quartiles (1.61, 2.39). Values above 7.4 have not been plotted. The boxplot suggests more coarse PM in AZ than in other locations and more variability in particle size distribution in OK.

Table S 8. Reference data sources and details

Site	$PM_{2.5}$ Data Source	$PM_{2.5}$ Method Ref	$PM_{2.5}$ Method Code	$PM_{2.5}$ Method POC	$PM_{2.5}$ Param Code	RH Data Source	Method Ref	RH Method POC Ref	RH Param Code Ref
AZ	AQS API	Thermo Scientific TEOM 1405-DF Dichot. with FDMS	182	3	88101	AirNow-Tech	Unspecified Reference	1	62201
CO	AQS API	Teledyne API T640	236	3	88101	AQS API	HYGROSCOPIC PLASTIC FILM	1	62201
DE	AQS API	Teledyne API T640	236	6	88101	AQS API	HYGROTHERMOGRAPH ELEC OR MACH AVG	1	62201
GA	AQS API	Teledyne API T640X	238	3	88101	AQS API	HYGROSCOPIC PLASTIC FILM	1	62201
NC	OAQPS AQAD AAMG (via envista)	Teledyne API T640X	238		88101	OAQPS AQAD AAMG (via envista)	R.M. Young 41382 VC		62201
OK	AQS API	Teledyne API T640	236	3	88101	AQS API	Vaisala 435C RH AT Sensor	2	62201
WI	AQS API	Teledyne API T640X	238	3	88101	IEM ASOS METAR Download, AQS API			

1.1.1 Arizona Site

The West Phoenix site (Figure S 2) was established in 1984. This Neighborhood scale SLAMS site is in an area of stable, high-density residential properties. This location monitors for CO, NO_2, O_3, PM_{10}, and $PM_{2.5}$. In addition, this is a QA colocation site for $PM_{2.5}$ where the Maricopa County Air Quality Department (MCAQD) operates one filter-based $PM_{2.5}$ FRM sampler along with one continuous $PM_{2.5}$ FEM analyzer as per 40 CFR Part 58 Appendix A. Meteorological monitors operating at this site include ambient temperature, barometric pressure, delta T (temperature inversion), and wind speed/direction. The sensor pods were deployed at this location on 8/12/2019. Sensors were installed on the shelter railing (Figure S 3, Figure S 4).

Table S 9. Additional Details AZ Site

Name (ID):	West Phoenix (AZ)
AQS ID:	04-013-0019
Address:	3847 W Earll Dr.<br>Phoenix, Arizona
Coordinates:	33.48385N, -112.14257W
Spatial Scale:	Neighborhood
Site Type:	Population Exposure<br>Highest Concentration

Table S 10. AZ Site Monitors

Criteria Pollutant	Reference Monitor Make - Model
CO	Teledyne – API 300T
NO_2	Teledyne – API 200T
O_3	Teledyne – API 400T
$PM_{2.5}$	Thermo – TEOM 1405-DF *(Primary)*<br>Thermo – Partisol 2025 *(Secondary)*<br>Teledyne -T640x
PM_{10}	Thermo – TEOM 1405-DF<br>Teledyne – T640X
Meteorological Parameters	Monitor Make - Model
Ambient Temperature (T)	MetOne
Relative Humidity (RH)	RM Young
Wind speed/direction (WS/WD)	RM Young
DT (10m - 2m Temperatures)	MetOne

*Not all data are available from AQS and AirNow Tech some must be retrieved directly from the monitoring agency.

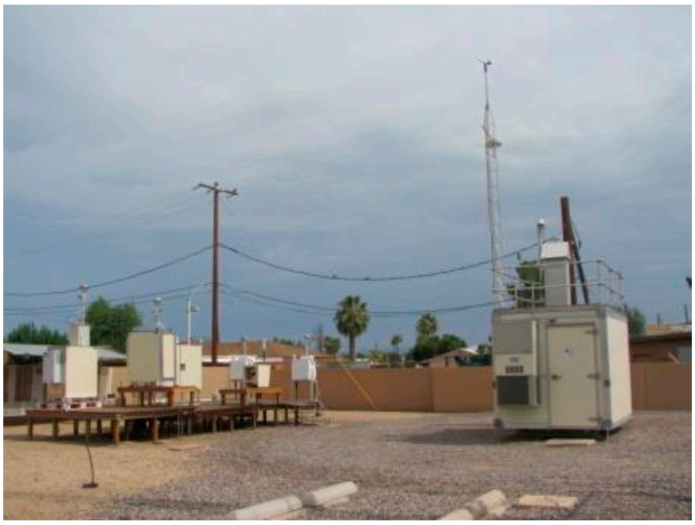

Figure S 2. Photo of AZ Site

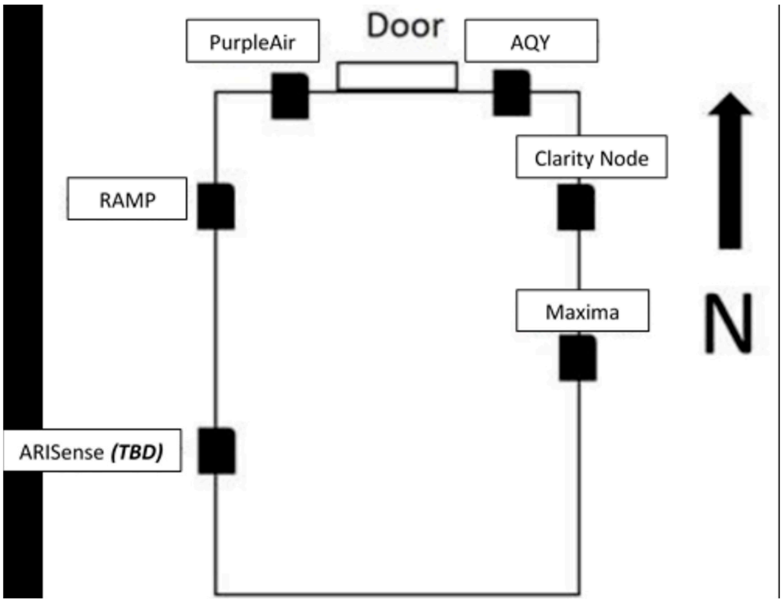

Figure S 3. AZ Site – Deployment diagram

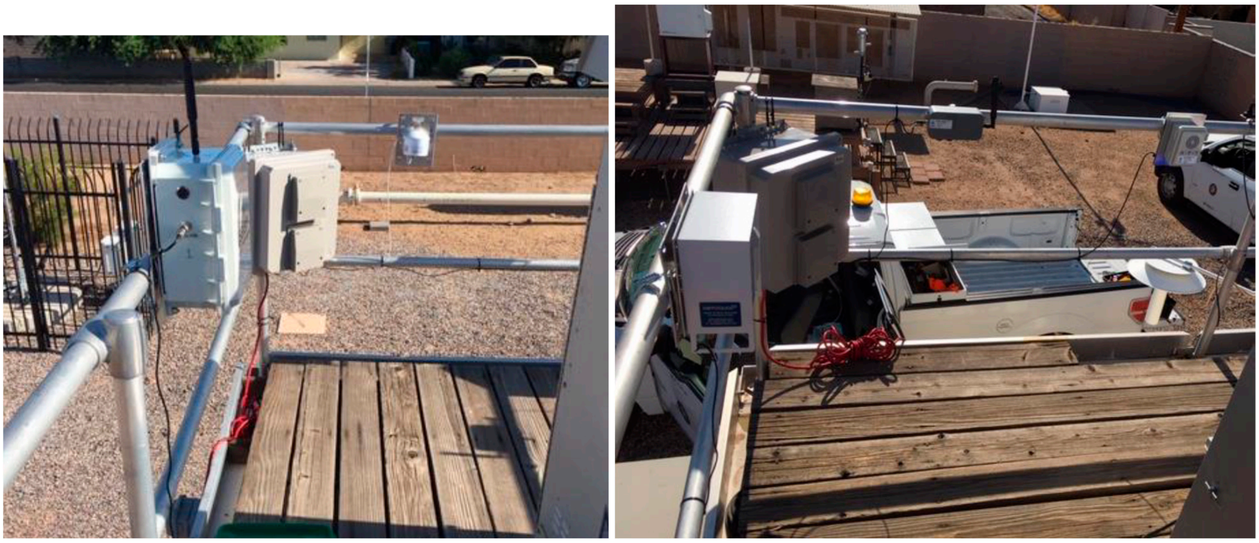

Figure S 4. AZ Site - Deployed Sensors. Left on railing RAMP, another sensor not in this study, PAR. Right on railing AQY, another sensor not this study, CNO, MAX.

1.1.2 Colorado Site

The La Casa site (Table S 11, Figure S 5) was established in January of 2013 as a replacement for the Denver Municipal Animal Shelter (DMAS) site when the DMAS site was decommissioned in late 2012 due to a land use change. The La Casa location in northwest Denver is a certified NCore site and includes a trace gas/precursor-level CO analyzer, and a NO_y analyzer, in addition to the trace level SO_2, O_3, meteorology, and particulate monitors. The site represents a population-oriented neighborhood scale monitoring area. The sensor pods were deployed on 7/31/2019 along the northwest corner of the railing atop the sampling shelter (Figure S 6).

Table S 11. CO Site Details

Name (ID):	La Casa (CO)
AQS ID:	08-031-0026
Address:	4587 Navajo St.<br>Denver, Colorado 80211
Coordinates:	39.779429 N, -105.005174 W
Spatial Scale:	Neighborhood<br>Urban
Site Type:	NCore<br>SLAMS

Table S 12. CO Site Monitors

Criteria Pollutant	Reference Monitor Make - Model
CO	Thermo – 48i-TLE *(trace)*
$NO/NO_2/NO_x$	Teledyne – API – 500U
NOy	Teledyne – API – 200EU *(trace)*
O_3	Teledyne – API – 400E
SO_2	Teledyne – API – 100EU *(trace)*
$PM_{2.5}$	Teledyne – T640<br>Partisol – 2025<br>GRIMM – EDM 180<br>MetOne SASS *(speciation)*<br>URG –3000N *(Carbon)*
PM_{10}	Partisol – 2025 *(colocated)*<br>GRIMM – EDM 180
Meteorological Parameters	Monitor Make - Model
Ambient Temperature (T)	MetOne
Relative Humidity (RH)	Climatronics
Wind speed/direction (WS/WD)	MetOne
Solar Radiation	Kipp & Zonen CMP-11

*Not all data are available from AQS and AirNow Tech some must be retrieved directly from the monitoring agency.

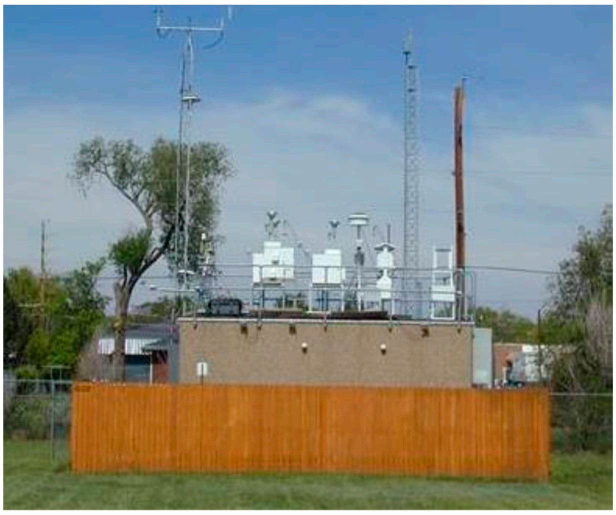

Figure S 5. Photo of CO Site

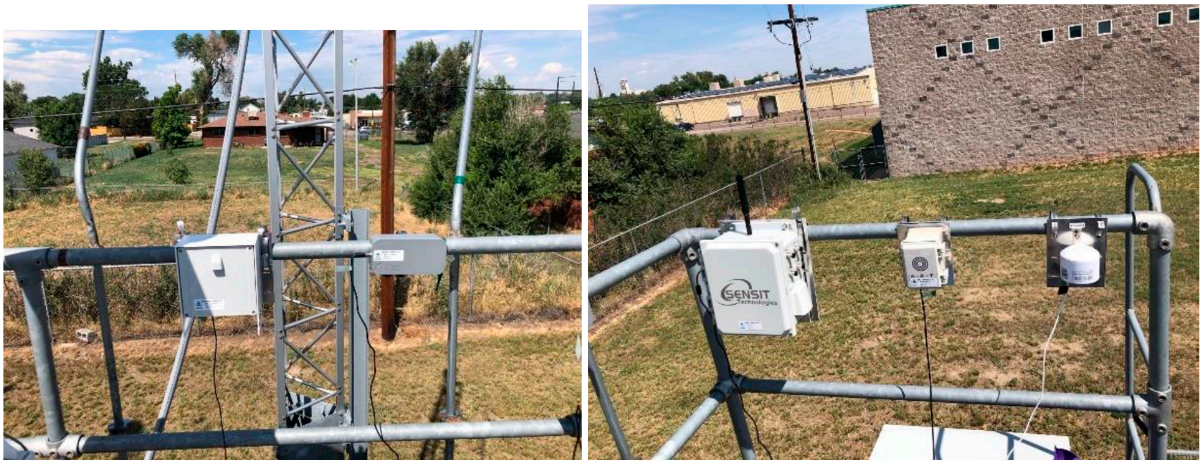

Figure S 6. CO Site – Deployed Sensors. Left AQY, Clarity. Right RAMP, Maxima, PurpleAir. (Arisense deployed later and not pictured)

1.1.3 Delaware Site

The MLK site (Table S 13, Table S 14, Figure S 7) is in Wilmington, Delaware at the intersection of Justison St. and MLK Blvd. In 1999, it replaced another urban site at 12th and King Streets which had operated at that location for over 20 years and was discontinued due to a change in land ownership. This site is the state NCore site and represents urban population exposure to multiple pollution sources. The sensor pods were deployed on 7/26/2019 on the railing of the northern corner atop the sampling shelter (see Figure 1 in the main text).

Table S 13. DE Site Details

Name (ID):	MLK (DE)
AQS ID:	10-003-2004
Address:	Justison St. & MLK Blvd, Wilmington, DE
Coordinates:	39.7395N, -75.5575W
Spatial Scale:	Neighborhood
Site Type:	Population Exposure<br>Maximum Concentration<br>NCore<br>PAMS

Table S 14. *MLK Site Monitors**

Criteria Pollutant	Reference Monitor Make - Model
CO	Teledyne – API – T300U
NO/NO_2/NO_x	Thermo – 42i-TL
NO/NOy	Thermo – 42i-Y
O_3	Thermo – 49i
SO_2	Thermo – 43i-TLE
$PM_{2.5}$	Teledyne – API – 640<br>Thermo – Partisol 2025i<br>SuperSass – URG 3000N *(Speciation)*
PM_{10}	Teledyne – API – 640 *(Continuous)*<br>Thermo – Partisol 2025i *(local conditions)*
Meteorological Parameters	Monitor Make - Model
Ambient Temperature (T)	Vaisala WXT-520
Relative Humidity (RH)	Vaisala WXT-520
Barometric Pressure (B_P)	Vaisala WXT-520
Wind speed/direction (WS/WD)	Vaisala WXT-520

*Not all data are available from AQS and AirNow Tech some must be retrieved directly from the monitoring agency.

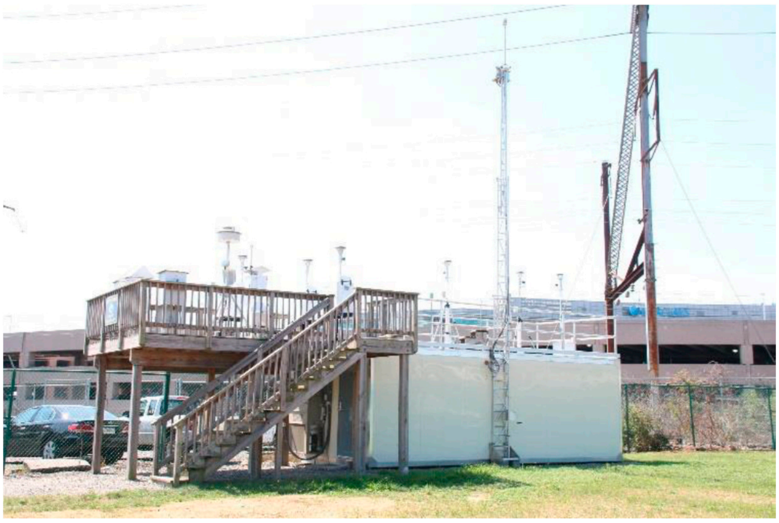

Figure S 7. Photo of DE Site

1.1.4 Georgia Site

The South DeKalb site (Table S 15, Table S 16, Figure S 8) was established as an O_3 site in Decatur, Georgia. Sensors were deployed on 8/1/2019 on the railing atop the sampling shelter (Figure S 9).

Table S 15. GA Site Details

Name (ID):	South DeKalb (GA)
AQS ID:	13-089-0002
Address:	2390-B Wildcat Road<br>Decatur, GA 30034
Coordinates:	33.6877N, -84.2905W
Spatial Scale:	Neighborhood
Site Type:	Population Exposure<br>Highest Concentration

Table S 16. South DeKalb Site Monitors

Criteria Pollutant	Reference Monitor Make - Model
CO	Thermo – 48i-TLE
$NO/NO_2/NO_x$	Thermo – 42i
NO/NOy	Thermo – 42iY
O_3	Thermo – 49i<br>Thermo – 49iPS
SO_2	Thermo – 43i-TLE
$PM_{2.5}$	Thermo – 2025<br>Teledyne – T640X<br>MetOne – SASS *(Speciated)*<br>URG – 3000N *(Speciated)*
PM_{10}	Teledyne – T640X<br>Shawnee Instruments
$PM_{10-2.5}$	Teledyne – T640X
Meteorological Parameters	Monitor Make - Model
Ambient Temperature (T)	RM Young – 41375VC
Relative Humidity (RH)	RM Young – 41375VC
Barometric Pressure (B_P)	RM Young – BP Sensor
Wind speed/direction (WS/WD)	RM Young – 05305vm

*Not all data are available from AQS and AirNow Tech some must be retrieved directly from the monitoring agency.

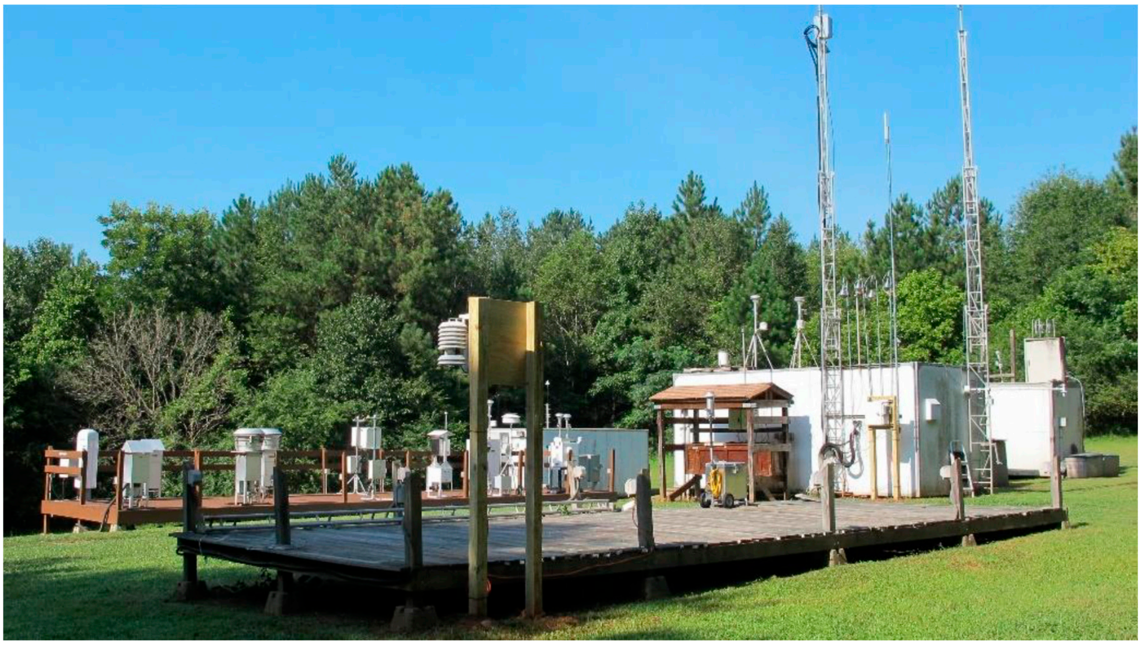

Figure S 8. Photo of GA Site

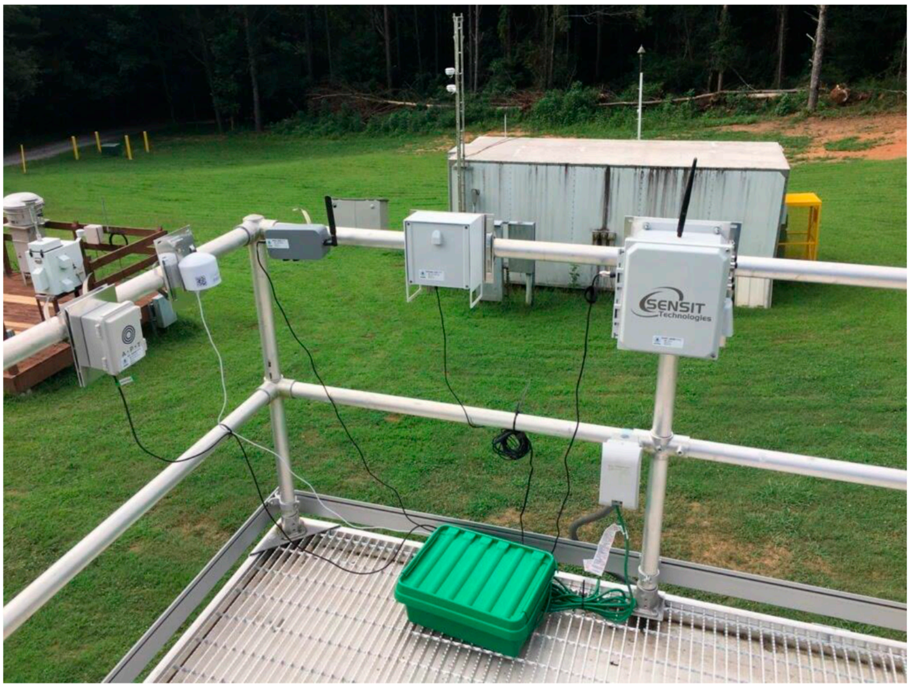

Figure S 9. GA Site – Deployed Sensors. On railing: Maxima, PurpleAir, Clarity, AQY, RAMP (Arisense deployed later and not pictured).

1.1.5 North Carolina Site

The Burdens Creek (AIRS) site (Table S 17, Table S 18, Figure S 10) is located on the U.S. EPA, RTP campus and is situated between Alexander Drive and Route 147. The site is intended to represent a neighborhood-scale

site as defined in *40 CFR Part 58, Appendix D*. OAQPS operates reference grade instruments in a free-standing shelter situated directly adjacent to the platform. Sensor pods were deployed on 8/1/2019 on a structure attached to a post near the north corner of the sampling platform (Figure S 11, Figure S 12). This site supports triplicate devices for the AQY, CNO, MAX, PAR and RAM sensor pods. In addition, seven ARS sensors were left colocated at the AIRS site.

Table S 17. NC Site Details

Name (ID):	AIRS (NC)
AQS ID	37-063-0099
Address:	111 TW Alexander Dr.<br>RTP, NC 27713
Coordinates:	35.889510N, -78.874572W
Spatial Scale:	Neighborhood
Site Type:	NCore

Table S 18. NC Site Monitors

Criteria Pollutant	Reference Monitor Make - Model
CO	Teledyne—API 300E
NO_2	Teledyne—API 500
O_3	Teledyne—API T265
BC	Magee—AE33
$PM_{2.5}$	Teledyne—API T640 and GRIMM
PM_{10}	Teledyne—API T640 and GRIMM
Meteorological Parameters	Monitor Make - Model
Ambient Temperature (T)	RM Young— 41382 VC
Relative Humidity (RH)	RM Young—41382 VC
Wind speed/direction (WS/WD)	Vaisala— WXT520

*Not all data are available from AirNow Tech some must be retrieved directly from the monitoring agency.

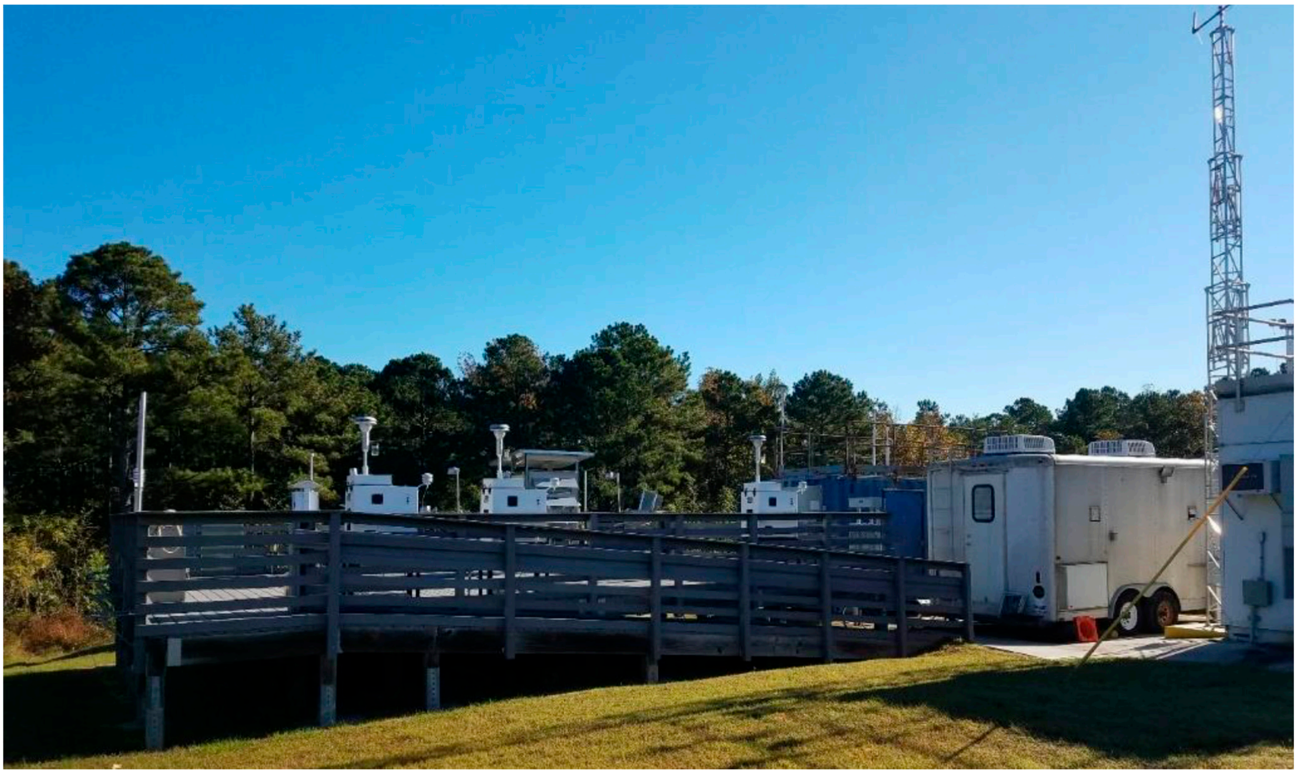

Figure S 10. Photo of NC Site. Sensors were deployed on the deck.

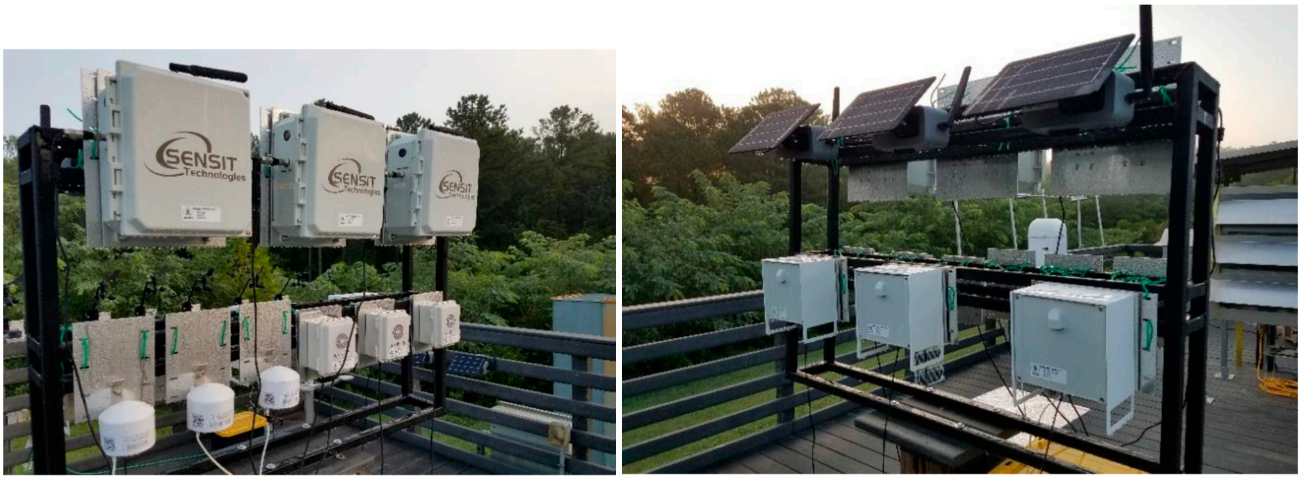

Figure S 11. NC Site – Deployed Sensors. Left top three RAMP, bottom three PurpleAir, three Maxima. Right top three Clarity Node-S (with solar power), bottom 3 AQY. Left and Right images show the front and back of the same structure where sensors were mounted.

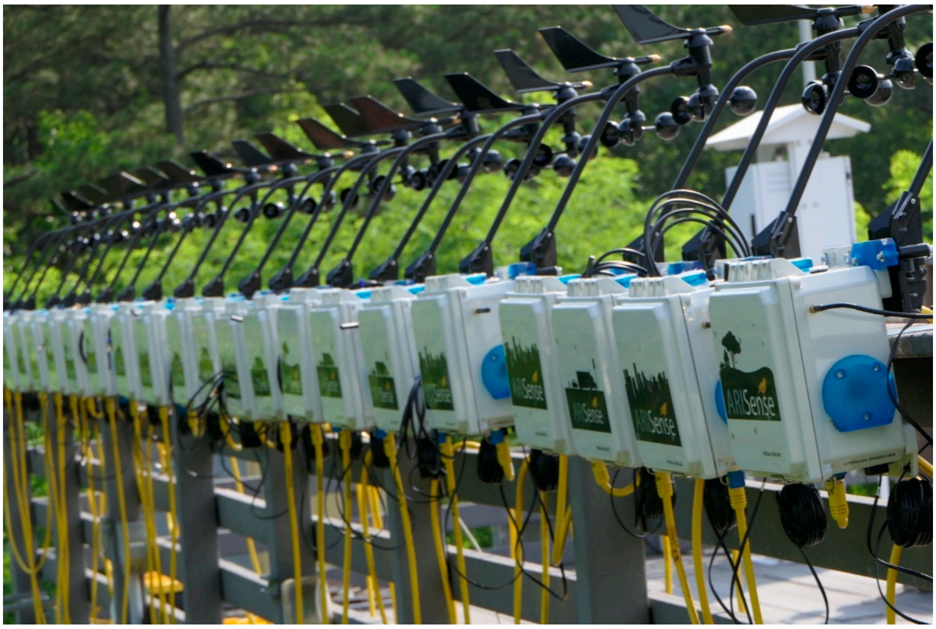

Figure S 12. Arisense sensors at AIRS. All sensors were run side-by-side but not all sensors pictured were used in this project.

1.1.6 Oklahoma Site

The Oklahoma Christian University monitoring site (Table S 19, Table S 20, Figure S 13) was established in the late 1970s. A containerized building houses the continuous instruments with a wooden platform directly adjacent for the filter-based instruments. Pollutants monitored include O_3, PM_{10} (filter based and continuous), $PM_{2.5}$ (filter based and continuous), $NO/NO_2/NO_x$, CO, SO_2 and meteorology. The site has solar panels to help defer the cost of power to the site. Sensors pods were deployed on 8/1/2019 on the metal railing of the sampling shelter (Figure S 14).

Table S 19. OK Site Details

Name (ID):	Oklahoma Christian University (OK)
AQS ID:	40-109-1037
Address:	2501 E. Memorial Rd<br>Edmond*, OK 73013
Coordinates:	35.614131N, -97.475083W
Spatial Scale:	Urban<br>Population Exposure
Site Type:	SLAMS

*Although Oklahoma Christian University has an Edmond, OK address, this monitoring site is officially located within the city limits of Oklahoma City, OK

Table S 20. OK Site Monitors

Criteria Pollutant	Reference Monitor Make - Model
CO	Teledyne –T300
NO_2	Teledyne – T200
NOx	Teledyne – T200
NO	Teledyne – T200
O_3	Teledyne – T400
SO_2	Thermo – 43IQ
$PM_{2.5}$	Teledyne – T640<br>Thermo – Partisol 2025i<br>MetOne SASS (*speciation*)<br>URG (*speciation*)<br>Tisch Hi-Vol
PM_{10}	Thermo – Partisol 2025i<br>Thermo – TEOM 1405<br>Teledyne – T640
Toxics	Atec
Meteorological Parameters	Monitor Make - Model
Ambient Temperature (T)	RM Young – 41382V
Relative Humidity (RH)	PM Young – 41382V
Wind speed/direction (WS/WD)	RM Young – 05305V

*Not all data are available from AQS and AirNow Tech some must be retrieved directly from the monitoring agency.

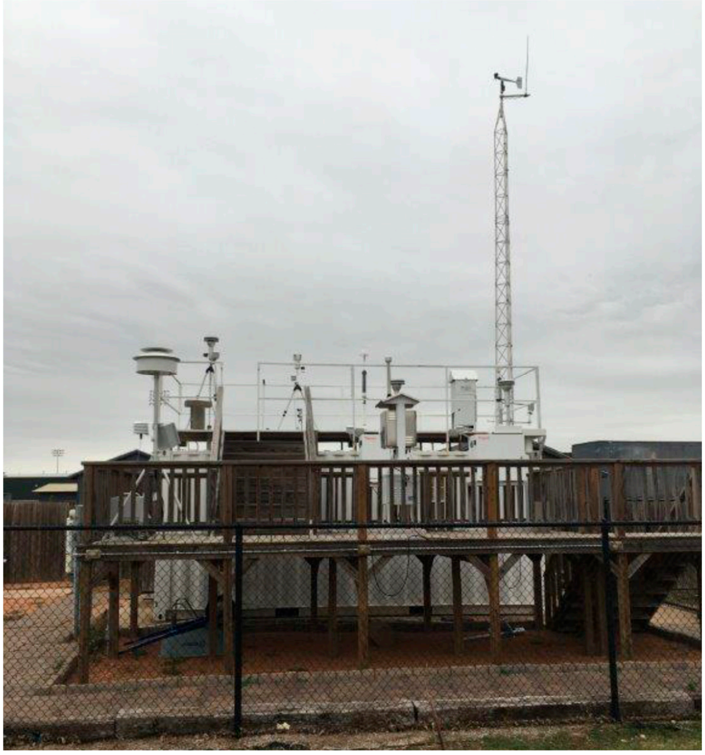

Figure S 13. OK Site

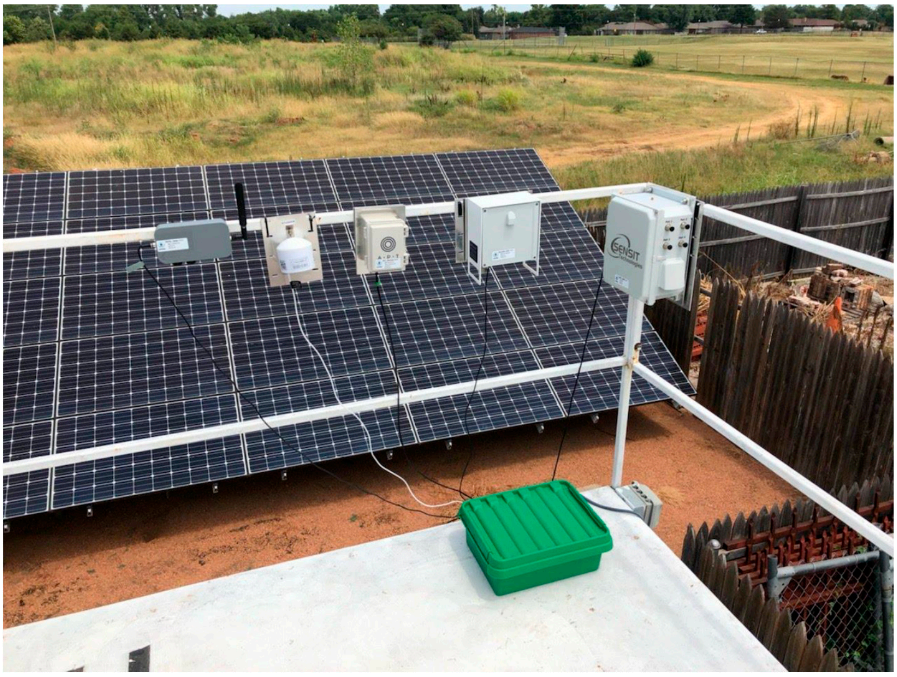

Figure S 14. Oklahoma Site – Deployed Sensors. On rail: Clarity, PurpleAir, Maxima, AQY, RAMP.

1.1.7 Wisconsin Site

The Wisconsin Department of Natural Resources (WDNR) Southeast Regional Headquarters urban site (Table S 19, Table S 20, Figure S 15) is in the WDNR parking lot at 2300 N. Martin Luther King Blvd. This site no longer exists; the building was shut down and the monitoring site was moved to a new location. Sample inlets are roughly 10 meters above ground level and 12 meters from the nearest road. Sensor pods were deployed on 7/22/2019 on the railing of the sampling shelter (Figure S 16).

Table S 21. WI Site Details

Name (ID):	Milwaukee Southeast Region Headquarters (WI)
AQS ID:	55-079-0026
Address:	2300 N. Dr. Martin Luther King Blvd.<br>Milwaukee, WI
Coordinates:	43.0610N, -87.9135W
Spatial Scale:	Urban<br>Neighborhood<br>Population Exposure
Site Type:	SLAMS

Table S 22. WI WDNR Headquarters Site Monitors

Criteria Pollutant	Reference Monitor Make - Model
$NO/NO_2/NO_x$ (NOy seasonal)	Teledyne – T200 (*will be replaced*)<br>Teledyne – T200U (*provides NOy seasonally*)<br>Teledyne – T500U (*will be installed*)
O_3	Teledyne – T400
SO_2	Teledyne –T100
$PM_{2.5}$	Teledyne – T640X
PM_{10}	Teledyne – T640X
$PM_{10-2.5}$	Teledyne – T640X
Meteorological Parameters	Monitor Make - Model
Ambient Temperature (T)	MetOne
Barometric Pressure (B_P)	MetOne – 092
Wind speed/direction (WS/WD)	MetOne – 010C/020C
Solar Radiation	MetOne – 094

*Not all data are available from AQS and AirNow Tech some must be retrieved directly from the monitoring agency.

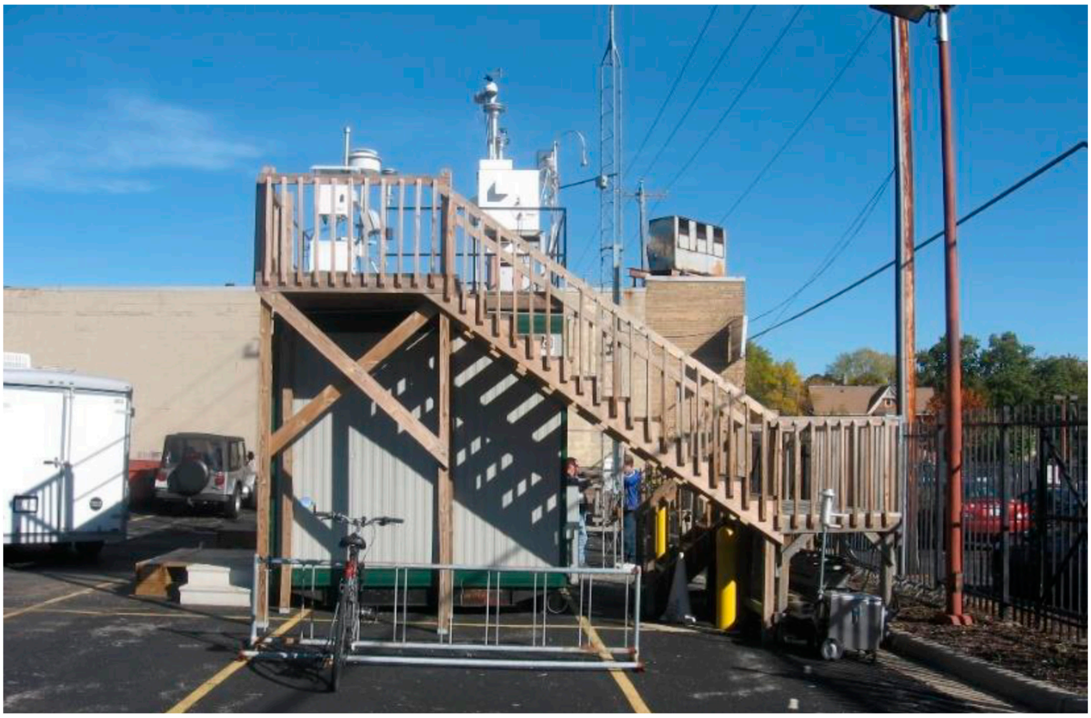

Figure S 15. Photo of WI Site

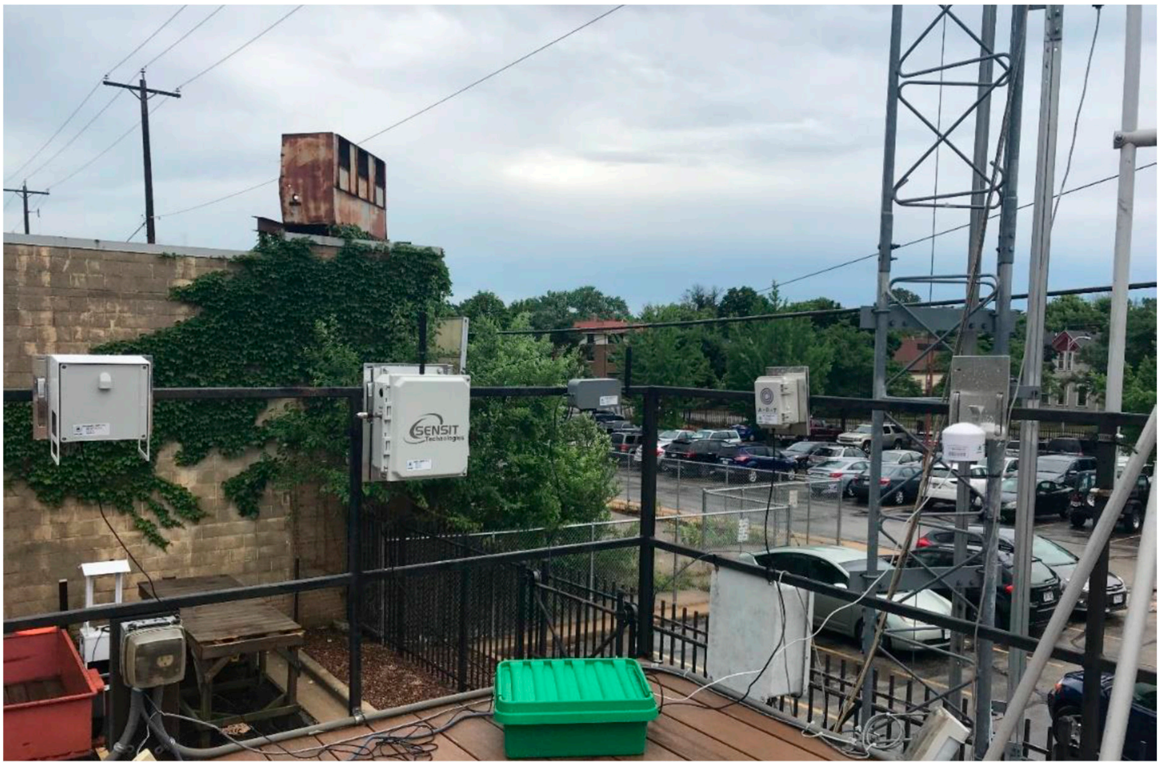

Figure S 16. WI Site – Deployed Sensors. On railing: AQY, RAMP, Clarity, Maxima, and PurpleAir (Arisense deployed later and not pictured).

1.2 Visual Inspections

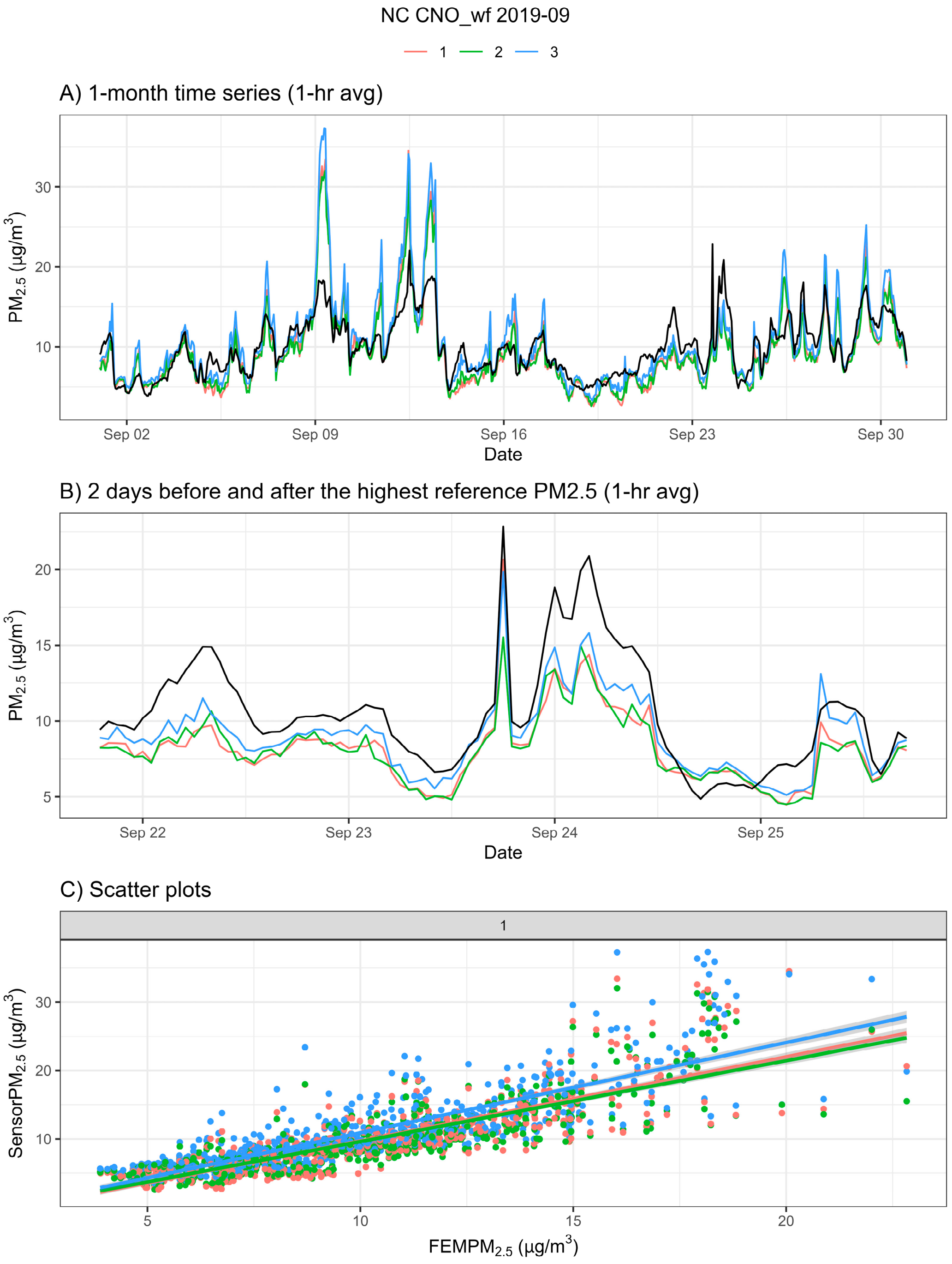

Figure S 17. Visual inspection of each sensor type, at each site, each month was used to identify any problems with time synchronization, outliers, or other issues. Example plot for Clarity wildfire corrected (CNO_wf) data at Research Triangle Park, NC site in September of 2019. The black line (A, B) represents monitor data.

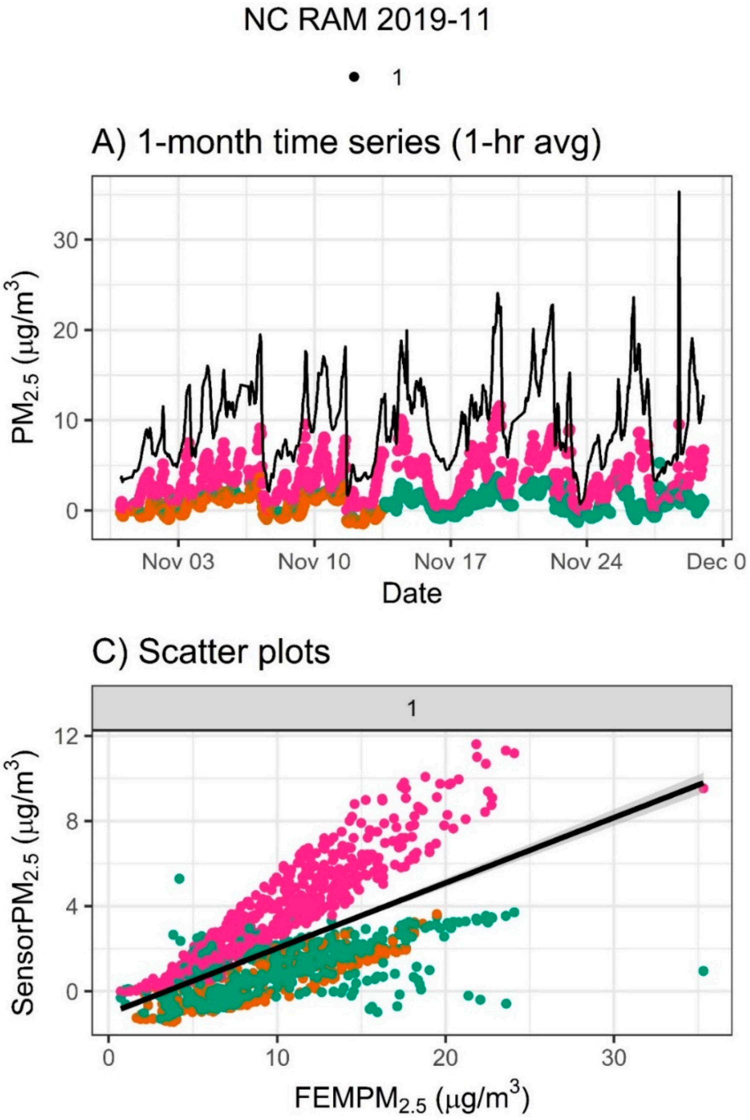

Figure S 18. An example of low precision between RAM sensors in NC.

1.3 Data Completeness, Common Failure Points, and Flagging

Several sensors arrived damaged during shipping. In general, the exteriors of the sensors were cushioned well but damage occurred when heavy internal components (e.g., pumps, batteries) were not robustly secured. This damage was quickly identified upon inspection from shipment. These sensors could be made more robust by adding support structures, additional internal support for shipping, isolation, and proper mounting of heavy components (e.g., metal brackets, secured to solid surfaces), and protection for delicate connections. Occasionally, wiring within some sensors became detached potentially due to vibration or jostling during transport in some cases it was very challenging to reattach due to the large number of connections. More secure connections and labeling of wires and components may make it easier for users to find and address issues after delivery. In most cases, damaged units were sent back to the manufacturer for repair and design improvements, but project staff assisted with some modifications.

The sensors had variable data completeness (Table S 23). Months with at least 80% of hourly averages as compared to the monitor data were considered complete. The AQY sensors had the highest completeness of $PM_{2.5}$ data with on average one incomplete month per sensor. The MAX and RAM were the next most complete with two incomplete months per sensor on average. The Clarity averaged three incomplete months per sensor and the PurpleAir averaged three and a half incomplete months per sensor. The ARS was the most incomplete with typically four months missing and these sensors were deployed for the shortest amount of time (typically 10 months instead of the 15 months on average for the other sensor types) due to their poor design and amount of work needed to get them operational. There was no clear trend of change in data completeness over time.

Data completeness is highly dependent on the frequency with which sensors are checked, the frequency of data review, and the time and expertise of those doing the troubleshooting. This is especially true for offline sensors (i.e., RAM) where missing data are identified once the data are manually downloaded. In multi-pollutant samplers, some pollutant measurements had lower completeness indicating a need to review data for all parameters measured. Often, one measurement would fail while other measurements were made as normal. More rarely, the failure of one component would interfere with the operation of the sensor package leading to no data collected from any component.

As previously mentioned, the deployment of the ARS units was delayed. This was largely due to the unavailability of parts, redesign of the sensors to incorporate a new PM sensor component, shipping damage, communication issues within the PM sensor causing communications to freeze, and the need for several firmware updates including one to remove a low temperature filter preventing data capture under freezing conditions. Troubleshooting these initial issues required several months of work that was not included in the flag total for the project.

Throughout the project we were in contact with manufacturers communicating common issues and failure points and many manufactures have taken these issues into consideration as they have improved the sensor design for more recent versions and models. All sensors except the ARS had acceptable data completeness for this project's objectives. Results from this project were somewhat delayed due to the more challenging than expected operation of the ARS. It is important for sensor users to understand the amount of staff time that may be needed to keep the sensors up and running to their desired degree. More time keeping sensors up and running is typically less time spent towards analyzing data and getting to the results of the project.

Table S 23. Months with <80% data completeness as measured by hours of sensor data/hours of monitor data. The sensors in GA were deployed for the longest at the request of the agency. AQY sensors were run for a few extra months since it was determined part way through the project that the gas sensors in some of them were unplugged leading to less usable data. The ARS were deployed for less time because of the multiple issues in making them operational.

	AQY	ARS	CNO	MAX	PAR	RAM	average
AZ	0	0	0	1	5	1	1.2
CO	2	7	5	2	2	1	3.2
DE	2	1	1	2	2	0	1.3
GA	2	9	4	4	5	3	4.5
NC*	4	**	0	1	4	1	2.0
OK	0	5	5	1	4	4	3.2
WI	0	2	5	5	5	5	3.7
total	10	24	20	16	27	15	18.7
average	1.4	4.0	2.9	2.3	3.9	2.1	2.8

*3 of each sensor type deployed value is the sum of incomplete months for all 3 sensors
**data incomplete. More than 7 sensors were deployed in NC and only the sensors with the best completeness were used so the incomplete months have not been reported as they would not be comparable to the other sensors.

Table S 24.. Flag Definitions.

Flag #	Definition	Parameter(s) Affected	Note
0	No flags/data errors known or reported.	ALL	Confirms that each file was checked by Jacobs staff and that no flags were noted during process review.
1	Sensor operating somewhere other than field site.	ALL	Identifies range of data, both Start/Stop timestamps required.
2	Warm-up Period.	ALL	Identifies range of data, both Start/Stop timestamps required.
3	Intentional shutdown for data collection/maintenance.	ALL	Identifies power off event, only Stop timestamp required.
4	Routine sensor maintenance.	ALL	Identifies range of data, both Start/Stop timestamps required. i.e. *CoolTerm reset time for the RAMP.*
5	Operator working near device (scheduled site visit).	ALL	Identifies range of data, both Start/Stop timestamps required.
6	Sampling Interval Abnormality.	ALL	Could identify a single point or range of data depending on the extent of the error.
7	Data Loss- User Error.	ALL	Identifies range of data, both Start/Stop timestamps required. Start/Stop timestamps will identify a datapoint that is not present in raw file.
8	Data Loss- Cellular/Wi-Fi Communication Error.	ALL	Identifies range of data, both Start/Stop timestamps required. Start/Stop timestamps will identify a datapoint that is not present in raw file.
9	Data Loss- Power Connection Error.	ALL	Identifies range of data, both Start/Stop timestamps required. Start/Stop timestamps will identify a datapoint that is not present in raw file.
10	Data Loss- Sensor Malfunction- Hardware.	ALL	Identifies range of data, both Start/Stop timestamps required. Start/Stop timestamps will identify a datapoint that is not present in raw file.
11	Data Loss- Sensor Malfunction- Firmware.	ALL	Identifies range of data, both Start/Stop timestamps required. Start/Stop timestamps will identify a datapoint that is not present in raw file.
12	Data Incomplete- Sensor Malfunction- Met Data.	T, RH, P, DP, WS, WD	Could identify a single point or range of data depending on the extent of the error. *List each parameter on separate row.*
13	Data Incomplete- Sensor Malfunction- Gas Data.	CO, CO_2, NO, NO_2, O_3	Could identify a single point or range of data depending on the extent of the error. *List each parameter on separate row.*
14	Data Incomplete- Sensor Malfunction- PM Data.	PM_1, $PM_{2.5}$, PM_{10}	Could identify a single point or range of data depending on the extent of the error. *List each parameter on separate row.*
15	Data Value- Drastic/Sudden spike or decrease.	ANY	Commonly assigned during data analysis of hourly averaged data.
16	Data Value- Questionable, outside of expect range.	ANY	Commonly assigned during data analysis of hourly averaged data. Context dependent but typically > 500 µg/m^3 for $PM_{2.5}$.
17	Data Value- Timestamp adjusted from DST to LST.	DTS	This would only be used for those sensors that don't already require UTC time conversion. i.e. RAMP.

Table S 25. Overall hours flagged by flag type (Table 3Table S 24.). Excluded flags with <24 hours flagged. Note that nine sensors running for a year yield 78,840 total hours or 3,285 days. The total data collected for each sensor type was variable due to a variety of factors. Only flags on $PM_{2.5}$ or "ALL" parameters were considered in this analysis since this paper focuses on $PM_{2.5}$ sensor performance.

Sensor.ID	Flag	total hours flagged (hours)	total hours flagged (days)
CNO	6	30643	1277
ARS	6	15614	651
PAR	9	13933	581
RAM	6	13426	559
PAR	6	10672	445
RAM	14	7447	310
MAX	10	5121	213
ARS	10	3780	157
ARS	14	3011	125
AQY	6	2995	125
AQY	10	2972	124
CNO	11	2635	110
MAX	9	2602	108
RAM	9	2389	100
ARS	9	1676	70
PAR	10	1170	49
AQY	9	1124	47
CNO	9	818	34
RAM	10	671	28
RAM	7	585	24
AQY	14	550	23
AQY	8	416	17
RAM	11	366	15
PAR	5	341	14
CNO	5	340	14
AQY	5	338	14
RAM	17	337	14
MAX	5	307	13
RAM	5	301	13
PAR	8	269	11
MAX	6	252	10
MAX	8	188	8
ARS	8	166	7
CNO	7	143	6
MAX	11	136	6
ARS	5	80	3
PAR	7	77	3
PAR	11	40	2

1.4 Hour of Day Performance

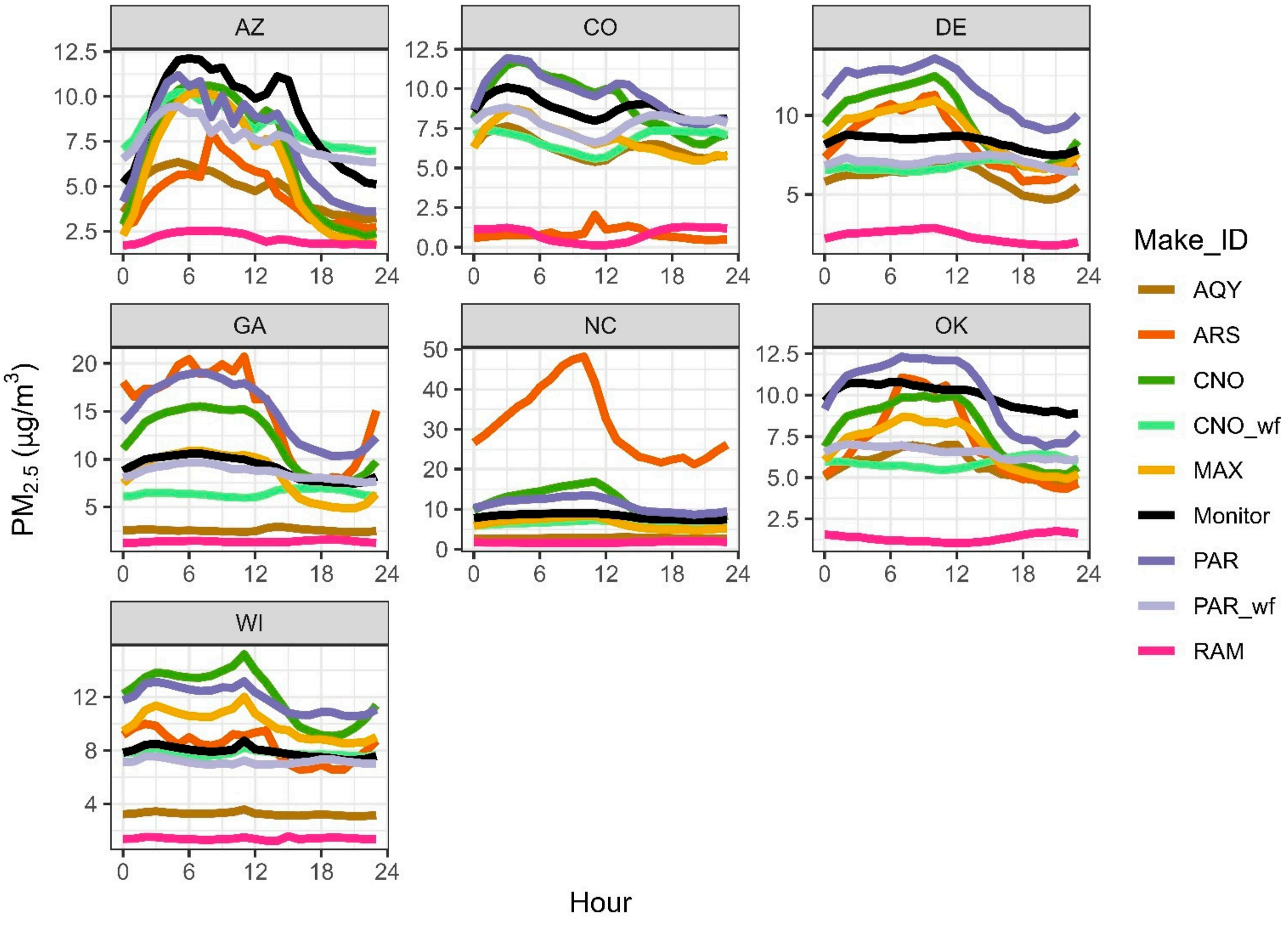

Figure S 19. Daily patterns of all sensors compared with the monitor (black).

1.5 Monthly Bias

Table S 26. Summary of MBE minimum, maximum, and the range of MBE by sensor make and location and R^2. Excludes months with <24 hour of data and pre and post-colocation.

						Uncorrected CNO and PAR			
Make_ID	Location	Min MBE	Max MBE	Range MBE	R^2	Min MBE	Max MBE	Range MBE	R^2
AQY	AZ	-14	-1	13	0.64				
AQY	CO	-4	-1	3	0.82				
AQY	DE	-6	0	6	0.75				
AQY	GA	-10	-3	7	0.42				
AQY	NC	-8	-3	5	0.41				
AQY	OK	-11	3	14	0.21				
AQY	WI	-7	-3	4	0.75				
ARS	AZ	-11	-1	10	0.45				
ARS	CO	-10	-7	3	0.15				
ARS	DE	-2	5	7	0.45				
ARS	GA	-5	8	13	0.12				
ARS	NC	-9	11	20	0.12				
ARS	OK	-13	0	13	0.42				
ARS	WI	-7	8	15	0.16				
CNO_wf	AZ	-6	1	7	0.88	-5	3	8	0.91
CNO_wf	CO	-7	-1	6	0.93	-3	5	8	0.79
CNO_wf	DE	-4	-1	3	0.81	-2	7	9	0.84
CNO_wf	GA	-6	-1	5	0.57	0	5	5	0.77
CNO_wf	NC	-6	1	7	0.6	-5	8	13	0.61
CNO_wf	OK	-6	-2	4	0.71	-6	1	7	0.68
CNO_wf	WI	-1	1	2	0.88	-2	13	15	0.83
MAX	AZ	-7	4	11	0.92				
MAX	CO	-6	1	7	0.92				
MAX	DE	-3	6	9	0.86				
MAX	GA	-3	2	5	0.79				
MAX	NC	-8	6	14	0.67				
MAX	OK	-8	0	8	0.55				
MAX	WI	-3	9	12	0.9				
PAR_wf	AZ	-6	1	7	0.87	-3	4	7	0.87
PAR_wf	CO	-4	0	4	0.94	-2	4	6	0.93
PAR_wf	DE	-4	2	6	0.84	0	13	13	0.86
PAR_wf	GA	-3	0	3	0.75	2	8	6	0.78
PAR_wf	NC	-5	2	7	0.76	-5	13	18	0.77
PAR_wf	OK	-9	-1	8	0.68	-6	3	9	0.67
PAR_wf	WI	-2	1	3	0.83	-2	8	10	0.81
RAM	AZ	-18	-3	15	0.88				
RAM	CO	-10	-4	6	0.35				
RAM	DE	-11	-4	7	0.58				
RAM	GA	-10	-6	4	0.64				
RAM	NC	-9	-3	6	0.32				
RAM	OK	-12	-7	5	0.38				

RAM	WI	-11	-4	7	0.45

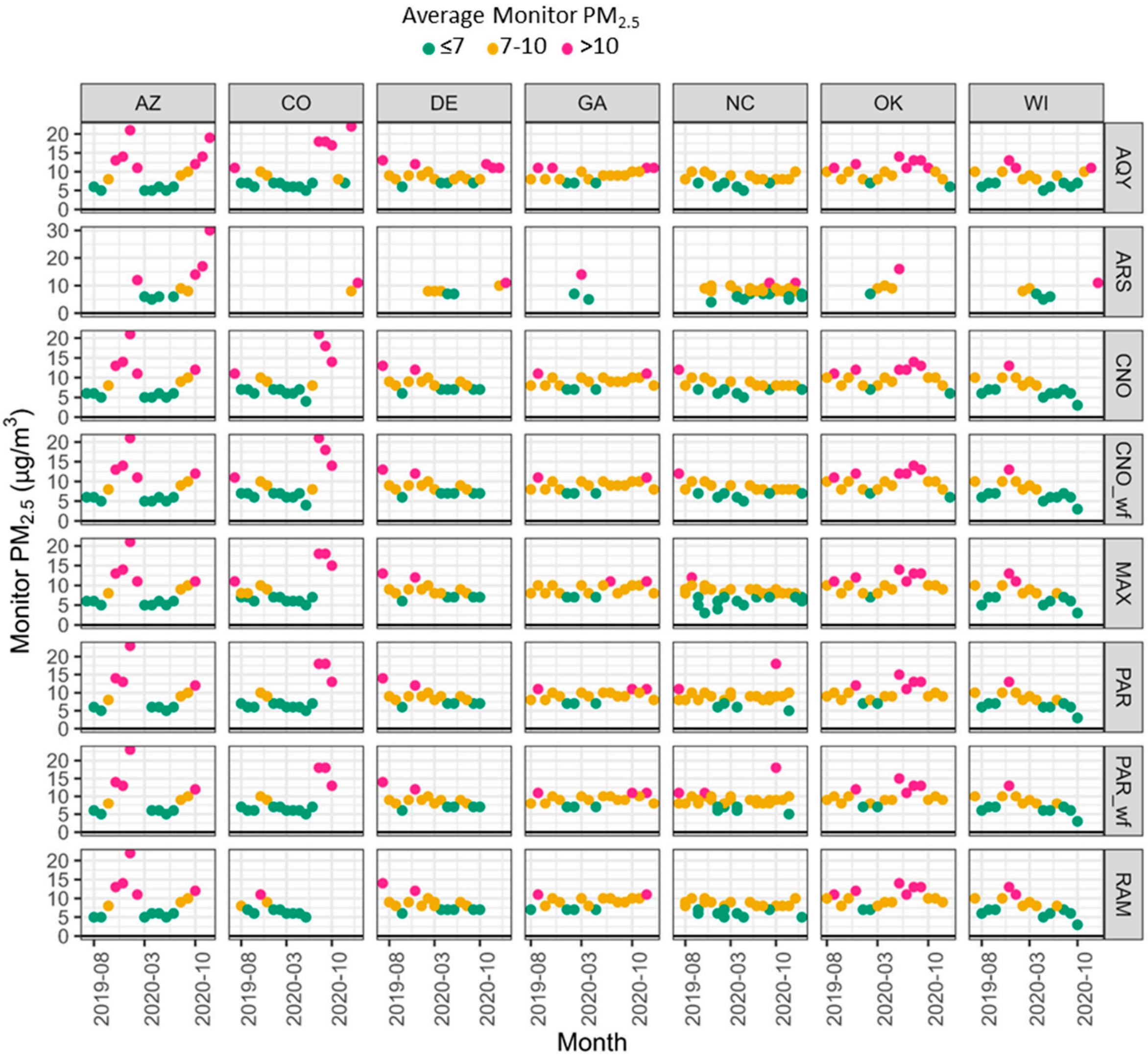

Figure S 20. Average FEM concentrations by month across locations. Sensors had different data completeness leading to slightly different average FEM concentrations.

Figure S 21 shows the difference in relationship between the sensors and the monitor during January 2020 and July 2020 to highlight seasonal differences in sensor performance six months apart. Most sensors in most locations show similar patterns. Some locations show much different concentrations during the two months (e.g., AZ, WI). Some sensors have more scatter during January (e.g., AQY CO, AQY DE, AQY OK, RAM NC) and others show lower estimates of $PM_{2.5}$ in January than in July at the same monitor concentrations (e.g., RAM DE, PAR_wf NC) or higher estimates of $PM_{2.5}$ in July than January at the same monitor concentrations (e.g., CNO AZ, AQY DE, AQY OK).

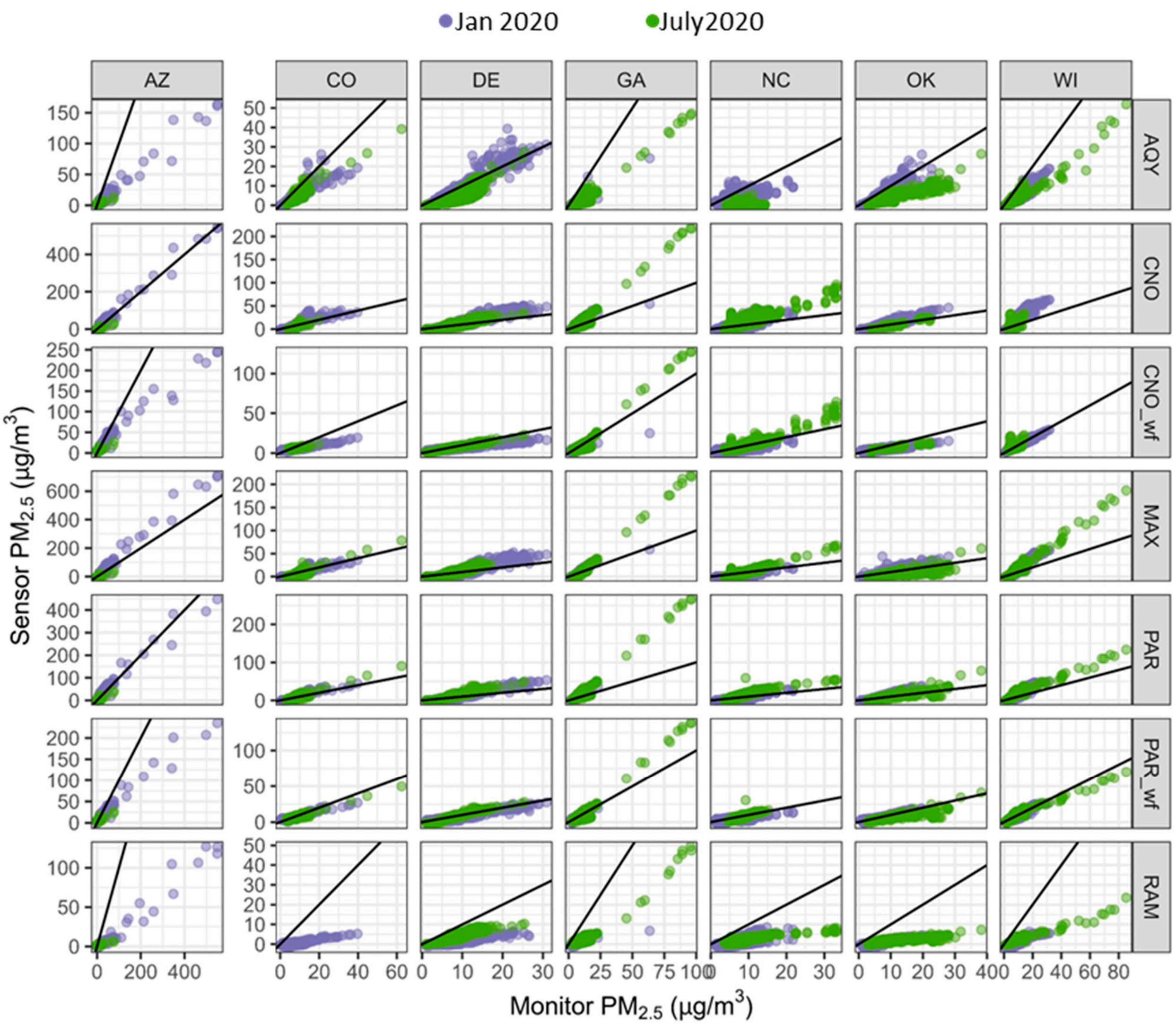

Figure S 21. Comparison of sensor performance in January and July of 2020. Note that ARS has been excluded because of low data completeness.
